# Case Report: Severe acute hypermagnesemia following inadvertent bath agent ingestion rapidly resolved by emergent hemodialysis

**DOI:** 10.3389/ftox.2026.1961156

**Published:** 2026-10-14

**Authors:** Chien-Chou Chen, Jun-Nan Chen, Tzu-Hsien Yang, Shih-Hua Lin

**Affiliations:** 1 Department of Internal Medicine, Tri-Service General Hospital Songshan Branch, National Defense Medical University, Taipei, Taiwan; 2 Division of Nephrology, Department of Medicine, Tri-Service General Hospital, National Defense Medical University, Taipei, Taiwan; 3 Division of Nephrology, Department of Medicine, Taichung Armed Forces General Hospital, Taichung, Taiwan

**Keywords:** bath agent, hemodialysis, hypermagnesemia, magnesium poisoning, toxicokinetics

## Abstract

Severe acute hypermagnesemia from excessive magnesium (Mg^2+^) exposure is rare but life-threatening and requires prompt recognition. We report a 54-year-old previously healthy man who presented to the emergency department with acute altered consciousness and generalized weakness. Physical examination showed stupor, hypothermia, hypotension, and generalized areflexia. Initial laboratory testing demonstrated mixed respiratory and metabolic acidosis, a normal serum creatinine concentration, and severe hypermagnesemia (serum Mg^2+^ concentration, 15.8 mg/dL). The initial 12-lead ECG demonstrated diffuse ST-segment depression, and an artificial intelligence-enabled electrocardiography (AI-ECG) model identified an estimated mortality risk above the 99th percentile. Nasogastric decompression retrieved minimal gastric contents, and initial treatment with intravenous calcium, saline, and a loop diuretic did not produce meaningful neurological improvement. Emergent hemodialysis resulted in rapid recovery of consciousness and a decrease in serum Mg^2+^ to 4.9 mg/dL during treatment, together with neurological improvement and a marked decline in AI-ECG-predicted mortality risk. Subsequent history-taking indicated inadvertent ingestion of approximately 100 mL of a private-label artificial hot-spring mineral preparation, for which spa personnel reported a nominal formulation of 30% magnesium chloride, 33% magnesium sulfate, and 10% potassium sulfate. He recovered without residual complications. This case emphasizes that a normal serum creatinine concentration at presentation does not preclude life-threatening magnesium toxicity after a large acute Mg^2+^ exposure. Timely hemodialysis should be considered when severe symptoms persist despite initial therapy.

## Introduction

Magnesium (Mg^2+^) is an important intracellular cation involved in energy metabolism, membrane stability, and neuromuscular conduction (Kröse and de Baaij, 2024; Morrison, 2023). Serum Mg^2+^ is normally maintained within a narrow range of 1.7–2.4 mg/dL through coordinated intestinal absorption, skeletal buffering, and renal excretion (Touyz et al., 2024). Among these mechanisms, renal excretion is the principal defense against Mg^2+^ accumulation, as urinary Mg^2+^ clearance can increase substantially in response to excess intake. Therefore, clinically significant hypermagnesemia is uncommon in individuals with preserved renal function. When hypermagnesemia occurs despite relatively normal renal function, it is usually related to excessive gastrointestinal Mg^2+^ exposure, such as Mg^2+^-containing laxatives, or less commonly to altered renal tubular handling of Mg^2+^ (Al Alawi et al., 2018). Nevertheless, renal protection may be overwhelmed by a sufficiently large or rapidly absorbed Mg^2+^ load, even before overt kidney dysfunction becomes apparent. In this clinical scenario, severe hypermagnesemia may develop rapidly and cause dose-dependent multisystem toxicity (Clark and Brown, 1992).

The clinical manifestations of hypermagnesemia correlate broadly with serum Mg^2+^ concentration. Mild hypermagnesemia may cause nausea, flushing, headache, lethargy, and depressed deep tendon reflexes, whereas moderate hypermagnesemia may progress to somnolence, absent reflexes, hypotension, bradycardia, and electrocardiographic abnormalities. Severe hypermagnesemia, particularly when serum Mg^2+^ exceeds 12 mg/dL, may result in fatal flaccid paralysis, apnea, respiratory failure, complete heart block, cardiac arrest, and coma (Clark and Brown, 1992; Haider et al., 2015; Cheungpasitporn et al., 2015; Broman et al., 2018; Si et al., 2024). In this report, we present a rare and instructive case of a 54-year-old man without known pre-existing kidney disease at presentation who developed severe acute hypermagnesemia after inadvertent ingestion of a concentrated bath preparation and recovered rapidly following emergent hemodialysis.

## Case description

A 54-year-old male was sent to the emergency department (ED) within 30 min of being found unresponsive at a health spa. His medical history was limited to treated hepatitis C and a remote appendectomy, with no known chronic illnesses or regular medication use. Upon arrival, his vital signs were notable for relative hypotension (104/58 mmHg), bradypnea (12 breaths/min), and hypothermia (34.3 °C), with a Glasgow Coma Scale (GCS) score of E3V2M5. Physical examination revealed a state of flaccid paralysis with generalized areflexia, including absent deep tendon reflexes.

Laboratory screening for common drugs of abuse was negative. Initial blood samples obtained after ED arrival showed blood urea nitrogen and serum creatinine concentrations of 14 and 0.9 mg/dL, respectively. Arterial blood gas analysis demonstrated mixed respiratory and metabolic acidosis, with a pH of 7.183, PaCO_2_ of 50.1 mmHg, and bicarbonate concentration of 18.4 mEq/L. Additional findings included a serum K^+^ concentration of 4.8 mmol/L, hyperchloremia of 116 mmol/L, an elevated measured serum osmolality of 324 mOsm/kg, a lactate concentration of 3.7 mmol/L, and a hemoglobin concentration of 18.1 g/dL. Serum Mg^2+^ was critically elevated at 15.8 mg/dL (reference range, 1.7–2.4 mg/dL). For the serial timeline, the time at which the initial laboratory results became available following ED arrival was designated as index time 0. The initial 12-lead ECG demonstrated sinus rhythm with prominent T waves and diffuse ST-segment depression (Figure 1A), and AI-ECG analysis identified a high estimated mortality risk (>99th percentile) (Lin et al., 2024). Non-contrast brain computed tomography was unremarkable.

**FIGURE 1:**
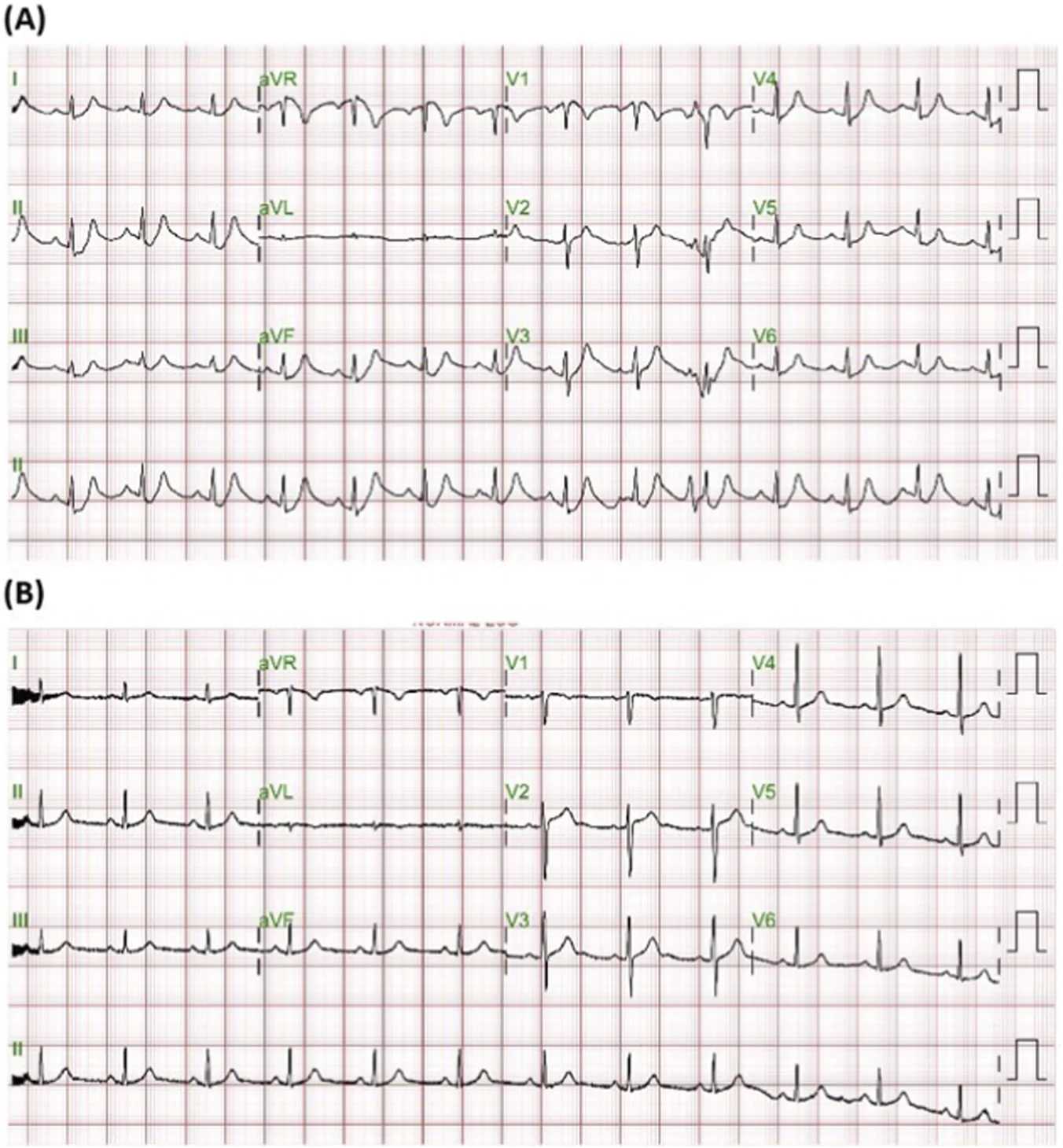
**(A)** ECG upon emergency visit. ECG on admission showed profound disruptions in cardiac ventricular repolarization, characterized by prominent, symmetrical T-waves and diffuse ST-segment depression, in the absence of classic PR interval prolongation or QRS widening. **(B)** ECG following resolution of hypermagnesemia. After emergent high-flux hemodialysis and subsequent saline and loop-diuretic therapy, the diffuse ST-segment depression resolved and T-wave morphology normalized.

No spontaneous vomiting or diarrhea was documented during the observed clinical course. A nasogastric tube was placed for gastric decompression, and aspiration yielded less than 10 mL of partially digested gastric contents. After the initial laboratory results became available (index time 0), intravenous calcium chloride was administered to antagonize magnesium-induced neuromuscular toxicity, followed by 1,000 mL of intravenous 0.9% saline and 40 mg of intravenous furosemide to promote renal Mg^2+^ elimination. Despite these measures, severe neurological depression persisted. Cumulative urine output from ED arrival to HD initiation was 400 mL.

Exposure history obtained from spa personnel during the acute investigation indicated that the patient had inadvertently ingested approximately 100 mL of a private-label artificial hot-spring mineral salt preparation used by the facility. Spa personnel reported a nominal formulation of 30% MgCl_2_, 33% MgSO_4_, and 10% K_2_SO_4_. The preparation was intended exclusively for external bathing and was not marketed under a consumer-facing commercial brand (Supplementary Figure).

After initial medical therapy, serum Mg^2+^ decreased from 15.8 mg/dL at index time 0–13.2 mg/dL in the pre-HD sample obtained at index +1 h, while the AI-ECG mortality risk remained above the 99th percentile. Given the persistent neurological and cardiopulmonary toxicity, emergent high-flux HD was initiated approximately 2 h after index time 0. HD was continued for 6 h, with a blood flow rate of 250 mL/min, a dialysate flow rate of 500 mL/min, a dialysate Mg^2+^ concentration of 0.5 mEq/L, and net ultrafiltration of 1,000 mL. Urine output during HD was 450 mL (mean, 75 mL/h). Blood pressure was 96/44 mmHg at HD initiation, 120/63 mmHg at the midpoint, and 145/80 mmHg at completion. One hour after HD initiation (index +3 h), the patient had regained full consciousness, with the GCS score improving from 10 to 15; serum Mg^2+^ had decreased further to 7.2 mg/dL, while the AI-ECG mortality risk remained above the 99th percentile. At index +5 and +7 h, serum Mg^2+^ decreased to 6.3 and 4.9 mg/dL, respectively, while the AI-ECG mortality risk percentile declined to the 83rd and 76th percentiles. The 6-h session ended at approximately index +8 h. At index +14 h, approximately 6 h after HD completion, serum Mg^2+^ showed a transient rebound to 6.2 mg/dL, whereas the AI-ECG mortality risk declined further to the 48th percentile, in parallel with resolution of diffuse ST-segment depression and reduced T-wave amplitude (Figures 1B, 2). The rebound hypermagnesemia was managed with continued volume expansion and diuretic therapy without further complications. Serum creatinine increased from 0.9 mg/dL at presentation to 1.3 mg/dL on day 2, consistent with a mild and transient acute kidney injury, and returned to 0.8 mg/dL by discharge. Blood urea nitrogen remained 14 mg/dL on day 2 and decreased to 12 mg/dL at discharge (Supplementary Table S1).

**FIGURE 2:**
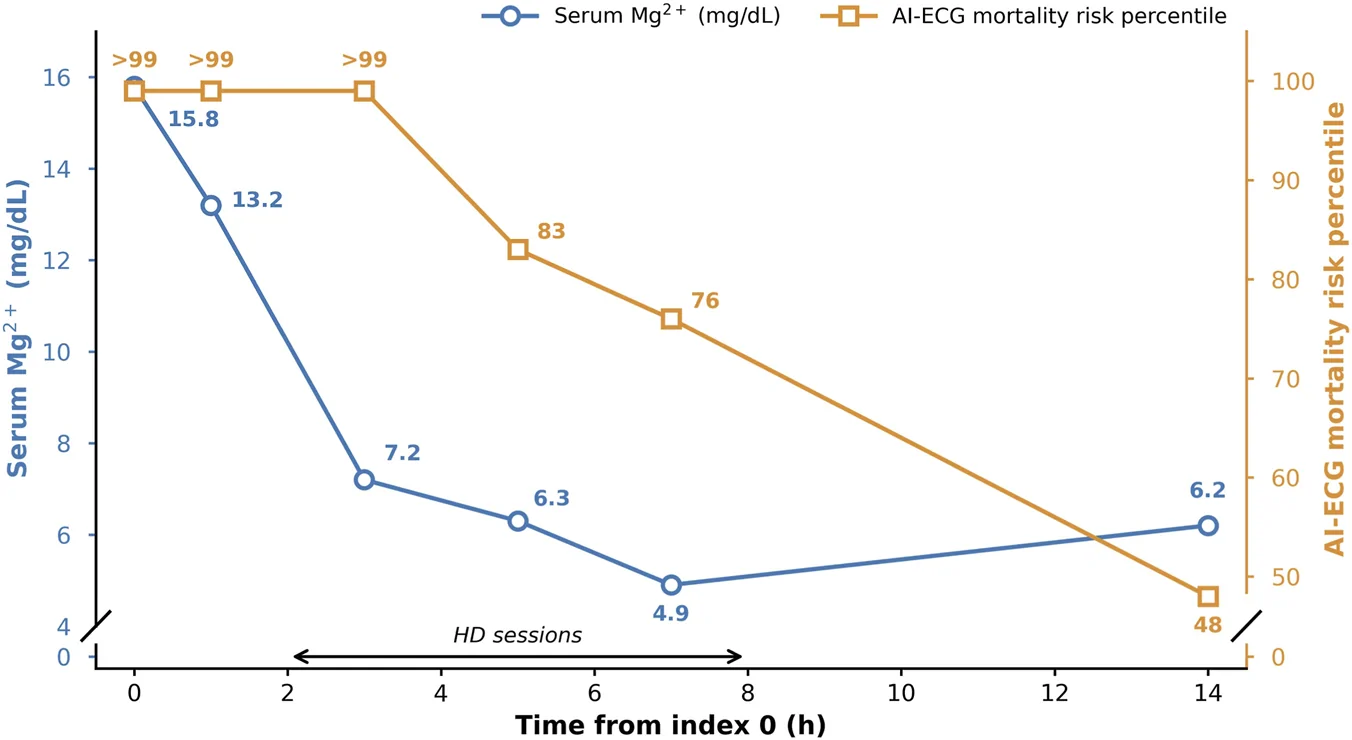
Temporal changes in serum Mg^2+^ concentration and AI-ECG mortality risk percentile. Index time 0 was defined as the time at which the initial laboratory results became available after ED arrival. Serum Mg^2+^ decreased from 15.8 mg/dL at index time 0–13.2 mg/dL at index +1 h, before HD initiation. HD began at approximately index +2 h and continued for 6 h, ending at approximately index +8 h. Serum Mg^2+^ subsequently decreased to 7.2, 6.3, and 4.9 mg/dL at index +3, +5, and +7 h, respectively, followed by a mild rebound to 6.2 mg/dL at index +14 h. The AI-ECG mortality risk remained above the 99th percentile at index 0, +1, and +3 h, then declined to the 83rd, 76th, and 48th percentiles at index +5, +7, and +14 h, respectively.

## Discussion

The patient developed acute altered consciousness, generalized areflexia, limb weakness, mixed respiratory and metabolic acidosis, and cardiovascular abnormalities after inadvertent ingestion of a Mg^2+^-containing bath preparation. Rapid neurological and biochemical improvement after high-flux hemodialysis supports consideration of early dialysis in severe symptomatic hypermagnesemia, even without known pre-existing kidney disease.

Renal excretion is the principal defense against Mg^2+^ accumulation, as increased filtered Mg^2+^ delivery and reduced tubular reabsorption usually compensate for excess intake (Dimke et al., 2023; De Baaij, 2023; Ellison et al., 2021; Costello et al., 2023). Exposure history obtained during the acute investigation indicated that the patient had ingested approximately 100 mL of a private-label artificial hot-spring mineral preparation with a reported nominal formulation of 30% MgCl_2_, 33% MgSO_4_, and 10% K_2_SO_4_. Although the original product documentation was unavailable and the precise elemental Mg^2+^ dose could not be confirmed, the serum Mg^2+^ concentration of 15.8 mg/dL, characteristic neuromuscular and cardiopulmonary manifestations, and rapid clinical and biochemical response to hemodialysis strongly supported a large acute gastrointestinal Mg^2+^ exposure.

Additional laboratory findings were compatible with exposure to a concentrated salt preparation but could not verify its individual components. Admission hyperchloremia and hyperosmolality supported a substantial solute load, whereas the serum potassium concentration was 4.8 mmol/L despite the reported nominal K_2_SO_4_ content. Serum potassium remained between 3.9 and 4.2 mmol/L during hemodialysis and subsequently increased to 5.3 mmol/L on day 2. These findings indicate that the full theoretical potassium load was not reflected in the extracellular potassium concentration at presentation. However, because serum potassium is influenced by gastrointestinal absorption, intracellular distribution, renal elimination, and subsequent treatment, it cannot be used to reconstruct the ingested potassium dose. Accordingly, these laboratory findings support exposure to a concentrated salt preparation but do not establish its precise composition or the absorbed doses of its individual constituents.

Severe hypermagnesemia after exogenous Mg^2+^ exposure appears to follow a convergent toxicokinetic pathway. Although reported sources include Mg^2+^-containing laxatives, cathartics, bowel preparations, enemas, antacids, supplements, obstetric Mg^2+^ therapy, and Mg^2+^-containing irrigation solutions (Table 1), clinically significant toxicity generally occurs when a large Mg^2+^ burden is coupled with factors that enhance absorption, prolong gastrointestinal exposure, disrupt mucosal barriers, or transiently reduce effective renal clearance (Clark and Brown, 1992; Walker et al., 2020; Bokhari et al., 2018). Prior severe cases in patients without advanced kidney disease have similarly clustered around bowel obstruction, visceral perforation, high-dose cathartic ingestion, or mucosal absorption as key toxicokinetic accelerators (Si et al., 2024; Walker et al., 2020; Mordes et al., 1975; McLaughlin and McKinney, 1998; Bansal et al., 2016) (Table 2). The present case extends this paradigm to a non-medical Mg^2+^-containing bath agent, demonstrating that concentrated nonmedical bath preparations may deliver a Mg^2+^ load sufficient to cause life-threatening toxicity even in the absence of advanced kidney disease. Accordingly, unexplained areflexia, flaccid weakness, altered consciousness, respiratory depression, hypotension, or conduction abnormalities should prompt clinicians to consider occult Mg^2+^ exposure, including nonpharmaceutical sources.

**TABLE 1:** Clinically relevant sources and routes of magnesium overdose.

Exposure source	Typical Mg compound	Route	Clinical setting/risk factor	Key clinical implication
Laxatives/cathartics	Magnesium citrate, hydroxide, sulfate, oxide	Oral	Constipation, repeated dosing, bowel obstruction, CKD, older age	Common overlooked source; check serum Mg in unexplained weakness, hyporeflexia, altered consciousness, or bradyarrhythmia
Bowel preparation agents	Magnesium citrate or sulfate	Oral	Volume depletion, elderly patients, CKD, delayed intestinal transit	Can combine Mg loading with dehydration, reducing renal Mg clearance
Magnesium enemas	Magnesium sulfate/Epsom salt	Rectal	Constipation, repeated enemas, pediatric or elderly patients, renal impairment	Rectal Mg exposure may cause abrupt systemic toxicity through rapid mucosal absorption
Non-Mg enemas after Mg cathartic use	Prior oral Mg oxide or Mg laxatives; enema may be glycerin-based	Oral accumulation + rectal intervention	Severe constipation, fecal retention, possible mucosal injury or ischemic colitis	Even Mg-free enemas may precipitate sudden Mg absorption if colonic Mg has accumulated
Antacids/supplements	Magnesium hydroxide, carbonate, oxide, citrate	Oral	Chronic use, CKD, older age, polypharmacy	Usually insidious; medication reconciliation is essential
Obstetric Mg therapy	Magnesium sulfate	Intravenous	Preeclampsia/eclampsia, oliguria, dosing error, renal dysfunction	Monitor reflexes, respiration, urine output, and serum Mg when toxicity is suspected
Mg-containing irrigation solutions	Magnesium-containing urinary or wound irrigation agents	Mucosal/urinary tract	Mucosal injury, urinary tract inflammation, obstruction, AKI	Rare but severe; systemic absorption may occur through injured mucosa

**TABLE 2:** Severe hypermagnesemia associated with exogenous magnesium exposure in patients without advanced kidney disease.

Author, year	Magnesium source	Baseline renal function	Clinical presentation	Initial serum Mg	Interventions	Clinical outcome
(Mordes et al., 1975)	Oral Magnesium citrate	Preserved *(SCr 2.0 mg/dL; Concomitant duodenal ulcer perforation)*	Lethargy, refractory hypotension	12.5 mEq/L (∼15.2 mg/dL)	Intravenous calcium, peritoneal dialysis	Survived (Hemodynamic stabilization and toxic clearance)
(McLaughlin and McKinney, 1998)	Oral Magnesium hydroxide *(Milk of Magnesia/Maalox)*	Preserved *(SCr 0.9 mg/dL; concomitant bowel obstruction)*	Confusion, abdominal distension, hypothermia, hypotension	9.1 mEq/L (∼11.1 mg/dL)	Intravenous calcium, saline resuscitation, furosemide, exploratory laparotomy	Fatal (Postoperative day 2 cardiac arrest)
(Walker et al., 2020)	Oral Epsom salt *(Magnesium sulfate)*	Preserved *(SCr 1.0 mg/dL)*	Generalized weakness, bradycardia, hypotension, coma, respiratory depression (requiring intubation)	18.2 mg/dL	Intravenous calcium, emergent hemodialysis	Survived (Rapid neurological awakening, no residual deficits)
(Si et al., 2024)	Oral Epsom salt *(Magnesium sulfate)*	Preserved *(SCr 0.94 mg/dL)*	Somnolence, generalized flaccid paralysis, generalized areflexia	15.42 mg/dL	Intravenous calcium, emergent hemodialysis	Survived (Electrolyte normalization and successful discharge)
(Bansal et al., 2016)	Intravesical/Renal pelvic irrigation *(Renacidin)*	Impaired (AKI) *(SCr 2.5 mg/dL)*	Complete neuromuscular paralysis, respiratory failure (requiring mechanical ventilation)	13.9 mEq/L (∼16.9 mg/dL)	Intravenous calcium, emergent intermittent and continuous hemodialysis (IHD/CVVH)	Survived (Complete restoration of motor and respiratory function)
Present Case	Inadvertent oral bath agent *(MgSO*_*4*_ */MgCl*_*2*_ *)*	Preserved *(SCr 0.9 mg/dL)*	Somnolence, hypothermia, generalized areflexia, mixed acidosis	15.8 mg/dL	Intravenous calcium, saline and loop-diuretic therapy, emergent high-flux hemodialysis	Survived (Neurological recovery 1 h after HD initiation; discharged on day 5)

Initial management of severe hypermagnesemia aims to antagonize magnesium-mediated toxicity and enhance renal elimination. Intravenous calcium may transiently counteract neuromuscular and cardiovascular depression, and saline-facilitated diuresis can increase urinary Mg^2+^ excretion when urine output is preserved; however, neither approach rapidly removes a large absorbed Mg^2+^ burden. Gastrointestinal decontamination was also limited in this case, as nasogastric aspiration retrieved less than 10 mL of partially digested fluid. Because magnesium salts are small inorganic ions and are not effectively adsorbed by activated charcoal, decontamination is mainly supportive and time-sensitive (Zellner et al., 2019). Persistent neurological and cardiopulmonary depression despite calcium administration, saline-facilitated diuresis, and limited gastrointestinal decontamination therefore supported the decision to initiate emergent high-flux hemodialysis.

Despite administration of 1,000 mL of intravenous saline, the patient’s GCS score remained 10 and the AI-ECG mortality-risk estimate remained above the 99th percentile before HD, raising concern for persistent magnesium-related cardiovascular toxicity and possible cardiac decompensation with further positive fluid accumulation. Net ultrafiltration of 1,000 mL over 6 h was therefore prescribed to offset the preceding saline load rather than to treat established volume overload. Despite an overall recorded fluid balance of approximately −850 mL, blood pressure increased from 96/44 to 145/80 mmHg as serum Mg^2+^ decreased and the neurological and electrocardiographic abnormalities improved, supporting magnesium-mediated cardiovascular depression as an important contributor to the initial hypotension.

During the 6-h HD session, serum Mg^2+^ decreased rapidly from 13.2 mg/dL in the pre-HD sample obtained at index +1 h to 7.2 mg/dL 1 h after HD initiation and to 4.9 mg/dL approximately 5 h after initiation, accompanied by rapid cardiopulmonary and neurological recovery. Although HD has been reported to reduce serum Mg^2+^ by approximately 30%–50% after 3–4 h, the observed clearance pattern may have been influenced by continued gastrointestinal absorption and tissue-to-serum redistribution. These findings support high-flux HD as an effective rescue therapy for severe symptomatic Mg^2+^ intoxication and underscore the importance of serial Mg^2+^ monitoring after large gastrointestinal exposures (Bansal et al., 2016; Misra and Nessim, 2017; Leenders et al., 2018; Bargnoux et al., 2024). The subsequent decline in AI-ECG mortality risk estimates alongside later biochemical and conventional ECG improvement should be interpreted as exploratory and supportive rather than diagnostic.

In conclusion, severe hypermagnesemia can occur after a large acute Mg^2+^ exposure even in patients without known pre-existing kidney disease and with a normal serum creatinine concentration at presentation. Serum Mg^2+^ should be measured in patients with otherwise unexplained neuromuscular, respiratory, cardiovascular, or consciousness disturbances when Mg^2+^ exposure is possible. Early hemodialysis should be considered when severe neurological or cardiopulmonary toxicity persists despite initial medical therapy.

## Data Availability

The original contributions presented in the study are included in the article/Supplementary Material, further inquiries can be directed to the corresponding author.
