# Supplementary material for: Case Report: Severe acute hypermagnesemia following inadvertent bath agent ingestion rapidly resolved by emergent hemodialysis
Source: Front Toxicol. 2026 Oct 14;8:1961156. doi: 10.3389/ftox.2026.1961156 (PMC13713025; doi:10.3389/ftox.2026.1961156)
Supplement: Supplementary file 1 [file Table1.docx]

Table S1. Serial clinical and laboratory findings throughout hemodialysis

| Parameter (unit) | Reference range | 0 h (initial lab results available) | +1 h  (pre-HD) | +3 h (HD +1 h) | +5 h (HD +3 h) | +7 h (HD +5 h) | +14 h (6 h post-HD) | Day 2 | Day 5 (discharge) |  |
| --- | --- | --- | --- | --- | --- | --- | --- | --- | --- | --- |
| ***Serum electrolytes and renal indices*** | | | | | | | | | | |
| Sodium (mmol/L) | 136 - 145 | 140 | - | - | - | 140 | 142 | 142 | 139 |  |
| Potassium (mmol/L) | 3.5 - 5.1 | 4.8 | - | 4.0 | 4.2 | 3.9 | 4.8 | 5.3 | 3.3 |  |
| Chloride (mmol/L) | 98 - 107 | 116 | - | - | - | 107 | 111 | 115 | 102 |  |
| Magnesium (mg/dL) | 1.7 - 2.4 | 15.8 | 13.2 | 7.2 | 6.3 | 4.9 | 6.2 | 4.7 | 2.1 |  |
| Total calcium (mg/dL) | - | - | - | - | - | - | - | - | 10 |  |
| Ionized calcium (mg/dL) | 4.5 - 5.3 | 5.16 | - | - | - | 4.56 | 4.48 | 4.7 | - |  |
| Blood urea nitrogen (mg/dL) | 8 - 20 | 14 | - | - | - | - | - | 14 | 12 |  |
| Creatinine (mg/dL) | 0.7 - 1.3 | 0.9 | - | - | - | - | - | 1.3 | 0.8 |  |
| Glucose (mg/dL) | 70 - 110 | 151 | - | - | - | 145 | 135 | 180 | 89 |  |
| Albumin (g/dL) | 3.5 - 5.0 | - | - | - | - | - | - | 4.7 | 4.2 |  |
| Measured osmolality (mOsm/kg) | 275 - 295 | 324 | - | - | - | 305 | - | - | - |  |
| Lactate (mmol/L) | 0.5 - 2.2 | 3.7 | - | - | - | 2.7 | 2.7 | 3.7 | 0.9 |  |
| ***Arterial blood gas*** | | | | | | | | | | |
| pH | 7.35 - 7.45 | 7.183 | - | - | - | 7.250 | - | - | 7.416 |  |
| PaCO₂ (mmHg) | 35 - 45 | 50.1 | - | - | - | 53.1 | - | - | 46.0 |  |
| PaO₂ (mmHg) | 80 - 100 | 281.1 | - | - | - | - | - | - | - |  |
| HCO₃⁻ (mEq/L) | 22 - 26 | 18.4 | - | - | - | 22.8 | - | - | 28.9 |  |
| ***Other laboratory data*** | | | | | | | | | | |
| White blood cells (×10³/µL) | 4.0 - 10.0 | 19.1 | - | - | - | - | - | 16.6 | 4.0 |  |
| Haemoglobin (g/dL) | 13.5 - 17.5 | 18.1 | - | - | - | - | - | 15.6 | 13.7 |  |
| Platelets (×10³/µL) | 150 - 400 | 262 | - | - | - | - | - | 197 | 169 |  |
| Creatine kinase (U/L) | 39 - 308 | 144 | - | - | - | - | - | 74 | 209 |  |
| AST / ALT (U/L) | < 40 / < 41 | 31 / 19 | - | - | - | - | - | 21/16 | 25/25 |  |
| Troponin I (pg/mL) | < 40 | 4 | - | - | - | - | - | 11 | 4 |  |
| C-reactive protein (mg/dL) | < 0.5 | < 0.1 | - | - | - | - | - | 7.15 | 1.75 |  |
| ***Bedside and monitoring*** | | | | | | | | | | |
| Glasgow Coma Scale  (E/V/M, total) | 15 | E3V2M5 (10) | E3V2M5 (10) | 15 | 15 | 15 | 15 | 15 | 15 |  |

Index time 0 was defined as the time at which the first laboratory results obtained after ED arrival became available. HD was initiated at approximately index +2 hours and continued for 6 hours, ending at approximately index +8 hours. -, not measured; ALT, alanine aminotransferase; AST, aspartate aminotransferase; ED, emergency department; HD, hemodialysis.
